# The association between statins and gait speed reserve in older adults: effects of concomitant medication

**DOI:** 10.1007/s11357-025-01682-x

**Published:** 2025-05-07

**Authors:** Anton De Spiegeleer, Antoon Bronselaer, Ine Mahieu, Dorien Vreys, Aaron Haslbauer, Jan-Philipp Leibfarth, Lara Van Schoote, Aster Wakjira, Mirko Petrovic, Evelien Wynendaele, Bart De Spiegeleer, Nele Van Den Noortgate, Reto W. Kressig, Roland Rössler

**Affiliations:** 1 Translational Research in Immunosenescence, Gerontology and Geriatrics (TRIGG) Group, Ghent University, Ghent, Belgium; 2 Department of Geriatrics, Ghent University Hospital, Ghent, Belgium; 3 Department of Internal Medicine and Paediatrics, Ghent University, Ghent, Belgium; 4 Department of Telecommunications & Information Processing, Faculty of Engineering and Architecture, Ghent University, Ghent, Belgium; 5 University Department of Geriatric Medicine FELIX PLATTER, Basel, Switzerland; 6 Jimma University, School of Pharmacy, Jimma, Ethiopia; 7 Drug Quality and Registration (DruQuaR) group, Faculty of Pharmaceutical Sciences, Ghent University, Ghent, Belgium; 8 University of Basel, Basel, Switzerland

**Keywords:** Physiological reserve, Gait, Statins, Polypharmacy, Real-world data (RWD), Geriatric population

## Abstract

Statins are frequently prescribed to older adults, yet their effects on ageing phenotypes such as frailty or physiological reserves remain poorly understood. Gait Speed Reserve (GSR), defined as the difference between maximal and usual gait speeds, serves as an indicator of physiological reserve, reflecting the body’s ability to perform beyond baseline functional levels. Polypharmacy, prevalent in this population, may contribute to inconsistent findings through interactions between statins and concomitant medications. We aimed to investigate how concomitant medications moderate the association between statin use and GSR in older adults. To this end, we conducted a cross-sectional observational cohort study using data from the Mobility Center at the University Department of Geriatric Medicine FELIX PLATTER, Basel, Switzerland (*n* = 5519 adults aged ≥ 60 years). Moderation regression analyses with propensity score weighting were used to evaluate the effect of concomitant medications on the association between statin use and GSR. Results showed statin use was associated with a lower GSR compared to non-use (− 1.9 cm/s [95% CI, − 3.1 to − 0.72]). However, ACE inhibitors and aspirin significantly influenced this association. The GSR difference for statin users compared to non-users increased by 3.7 cm/s (from − 2.2 to 1.5 cm/s; 95% CI, 0.0 to 7.4) with concomitant ACE inhibitor use and by 5.8 cm/s (from − 3.4 to 2.3 cm/s; 95% CI, 2.5 to 9.1) with aspirin use. We found no statistically significant association between statin use and usual gait speed, the secondary outcome. In conclusion, ACE inhibitors and aspirin interacted with statins, reversing the negative association with GSR into a positive one when co-used. Future clinical trials are needed to determine causality and further investigate the impact of concomitant medication use on statin effects in aging populations. Meanwhile, our findings underscore the importance of considering concomitant medication use when assessing the effects of statins in older adults.

**Supplementary Information:**

The online version contains supplementary material available at 10.1007/s11357-025-01682-x.

## Introduction

The global population of adults aged 65 and older is rapidly increasing, projected to rise from 1 billion in 2019 to 2.1 billion by 2050 [1]. However, ageing is a highly variable process, broadly categorized into healthy and accelerated ageing. Accelerated ageing is characterized by a fast decline in physiological reserves—the body’s maximal capacity to function beyond baseline levels [2]. Physiological reserve can be considered an early marker of functional capacity, with its decline contributing to reduced intrinsic capacity—a broader, higher-order concept—and the development of frailty, which is often positioned at the lower end of the physiological reserve spectrum [3, 4]. To ensure that current and future generations maintain a high quality of life in old age at acceptable healthcare costs, mitigating the decline in physiological reserves and slowing the progression of accelerated ageing is essential [5, 6].

Gait speed reserve (GSR), the difference between maximum and usual gait speed, is a valuable clinical measure of physiological reserve. It reflects an individual’s “gait performance plasticity,” which is critical for everyday tasks, responding to environmental challenges (e.g., crossing a street while the pedestrian signal is green), and maintaining independent living [7]. Aligned with its role as an indicator of physiological reserve, which may precede changes in intrinsic capacity and/or frailty status, GSR has been shown to effectively identify frail older adults and to be a strong predictor of critical clinical outcomes, including hospitalization and all-cause mortality [8–12]. Moreover, compared to static gait performance metrics such as usual gait speed—often used in frailty and intrinsic capacity assessments—GSR frequently exhibits earlier and more sensitive changes [11, 12].

Currently, there are no FDA- or EMA-approved pharmacological treatments countering accelerated ageing or restoring reduced physiological reserves [5, 17]. While research is ongoing to develop gero-protective drugs, the traditional drug discovery process is time-consuming and costly. A promising alternative is drug repurposing, which involves finding new therapeutic applications for existing medications [18]. Among potential gero-protective agents, statins are of particular interest. While primarily prescribed for secondary cardiovascular prevention due to their cholesterol-lowering properties, statins also exhibit pleiotropic effects. Preclinical studies, both in vitro and in vivo, have demonstrated statins’ beneficial effects on different hallmarks of ageing such as inflammation, epigenetics, telomere attrition, cellular senescence, mitochondrial dysfunction, and dysbiosis [19–23]. Large clinical trials have further highlighted statins’ potential gero-protective effects. In the JUPITER study, rosuvastatin reduced not only cardiovascular events but also high-sensitivity C-reactive protein (hs-CRP) levels in healthy older adults (median age 66) with elevated hs-CRP but without hyperlipidemia. Similarly, the REPRIEVE study demonstrated that pitavastatin lowered oxidation biomarkers, arterial inflammation, as well as cardiovascular events in middle-aged HIV patients (median age 50) [24, 25].

Although no study has yet investigated the association between statins and GSR or physiological reserve, several studies have examined the effects of statin use on frailty or gait metrics—though not explicitly framed as indicators of functional ageing—yielding inconsistent results. For instance, Dumurgier et al. analyzed the association between usual gait speed over time and statin use in the Three-City Study, a cohort of community-dwelling adults aged 65 and older from three French cities (Bordeaux, Dijon, and Montpellier). Including over 4000 participants and controlling for demographic and health-related factors, the study found that statin users experienced a slower decline in gait speed over 10 years [26]. In contrast, Gray et al., in the Women’s Health Initiative Study, found no significant association between statin use and timed 6-m walk performance in 5777 women aged 65 to 79 [27]. Beyond variability in gait performance measures, differences in population characteristics, statin types, and doses are mentioned as possible contributions to the inconsistency. Notably however, none of these studies have comprehensively examined the potential moderating role of concomitant medications on statin effects.

Given that older adults often take multiple medications, understanding these interactions is critical for accurately assessing the impact of statins on functional ageing. This study aims to address this gap by investigating how commonly used concomitant medications influence the association between statin use and gait speed reserve, a gait metric that serves as a measure for physiological reserve. Understanding the direction and magnitude of these effects will be crucial for balancing the risks and benefits of statin use in older populations.

## Methods

### Study design

We conducted a retrospective observational study using pseudonymized data from the Basel Mobility Center (FELIX PLATTER Hospital, Switzerland), including all first-visit patients between January 1, 2008, and May 30, 2023. Eligible participants were men and women aged 60 years or older who had attended the Basel Mobility Center during this period. For individuals with multiple visits, only data from the first visit were included in the analysis. Participants were excluded if data on usual or maximum gait speed were missing or incomplete at the first visit, or if medication information was unavailable or incomplete. The Basel Mobility Center is a specialized geriatric clinic for individuals referred due to mobility impairments, cognitive concerns, recurrent falls, or for preventive functional evaluations. Pseudonymized data were used with approval from the regional Ethics Committee (Ethikkommission Nordwest- und Zentralschweiz EKNZ, 2024–01296), in accordance with the Declaration of Helsinki guidelines. The requirement for informed patient consent was waived given the retrospective observational character of the study. This report adheres to the Strengthening the Reporting of Observational Studies in Epidemiology (STROBE) guidelines for cross-sectional studies [28].

### Exposure

Twenty-two drug classes were selected based on their significant contribution to chronic polypharmacy in older adults [29–31]. Medication use was determined through textual descriptions of recent medical reports and was cross-validated during patient visits via direct interviews conducted by trained hospital staff at the Basel Mobility Center. This dual-source approach ensured a structured, standardized assessment of medication use and minimized the potential for recall bias. The included drug classes were statins, 5α-reductase inhibitors, angiotensin-converting enzyme (ACE) inhibitors, aldosterone antagonists and other potassium-sparing agents, α-adrenergic antagonists, antipsychotics, aspirin, benzodiazepines, beta-blockers, biguanides, dihydropyridines, insulins or analogues, opioids, proton-pump inhibitors, sartans, selective serotonin reuptake inhibitors (SSRIs), sulphonamide loop diuretics, sulphonamide thiazide-likes, sulphonylureas, tricyclic antidepressants, thyroid hormones, and uric acid inhibitors. Drug intake was interpreted as long-term exposure, reflecting the stable nature of the Basel Mobility Center’s patient population, which generally consists of adults without acute conditions or short-term medication use. Furthermore, the 22 drug classes are predominantly used as chronic treatments. The textual medication descriptions included both generic and brand names of the drugs. Patients with missing medication history were excluded from the analysis. To convert the textual records into structured data, an index was constructed mapping Anatomical Therapeutic Chemical (ATC) codes to the corresponding drug names. This index incorporated the official ATC classification as well as an expanded index linking ATC codes to brand names, comprising a total of 19258 unique entries. To extract structured data, the medication history was subdivided into substrings using a regular expression (regex) split method. The resulting substrings were then matched against the drug names in the index through a two-step process [32, 33]. First, exact matches were attempted. If no exact match was found, a second step applied a two-level string comparator, where matches were either (i) exact if one of the tokens had fewer than four characters or (ii) based on the Damerau-Levenshtein metric for longer tokens [34]. An approximate match was accepted if the string similarity score was ≥ 0.85 and no surrounding negating expressions (e.g., “patient does not take…”) were detected. In the rare case of multiple approximate matches, the one with the highest similarity score was retained. Once a successful match—either exact or approximate—was established, the patient ID was linked to the corresponding ATC code. The quality of this automated annotation process was evaluated by having two independent physicians manually review a random sample of 80 participant records, verifying that the assigned ATC codes accurately reflected the medication history. The total accuracy was 93% (5% participants with a false positive ATC code, 2% participants with a false negative ATC code).

### Outcome

The primary outcome of this study was gait speed reserve (GSR), defined as the difference between usual gait speed and maximum gait speed. Usual gait speed was considered a secondary outcome. Gait analyses were conducted in accordance with the European guidelines for spatio-temporal gait analysis in older adults, utilizing the GAITRite® Platinum System, a carpet-based walkway approximately 10 m in length (CIR Systems, PA, USA) [35]. Gait data were processed using the proprietary GAITRite® software. To ensure accurate measurement, the walkway was equipped with 1.25-m optically identical but electronically inactive sections at both ends, designed to capture acceleration and deceleration phases. Participants wore their regular footwear during testing. Prior to gait analysis, standardized verbal instructions and a visual demonstration of the procedure were provided by the test administrator. No practice trials were conducted. For usual gait speed, participants were instructed to “walk at a normal, comfortable pace,” whereas for maximum gait speed, the instruction was to “walk as fast as you can without running.” Participants with missing gait data were excluded from the analysis.

### Covariates

Age, sex, body mass index (BMI, calculated as weight in kilograms divided by height in meters squared), physical activity levels (dichotomous, with physical activity being defined as engaging in moderate physical activity at least once a week), use of walking aids (dichotomous), and history of falls within the previous 12 months (dichotomous) were collected as covariates in a structured format. To ensure data consistency, nominal and categorical attributes were checked for uniformity of values, and numerical variables underwent outlier analysis through visual inspection of box plots and histograms (using Sturges’ binning). Any outlier values that were biologically implausible were manually reviewed and removed if deemed impossible (*n* = 4 for BMI).

To identify comorbidities linked to both statin use (exposure) and gait performance (outcome), medical histories were analyzed for the following conditions: hyperlipidemia, hypertension, diabetes, coronary artery disease, congestive heart failure, vascular disease (stroke, carotid stenosis or peripheral artery disease), chronic kidney disease, pulmonary disease, hip/knee osteoarthritis, Parkinson’s disease, low back pain, and depression. As comorbidities were recorded as free-text entries rather than in structured format, an index was created similar as for medication history, mapping both German and English terms for each condition. Unlike medication data, which could be parsed more directly, comorbidity descriptions required a “moving window” approach, iterating over all tokens in the patient’s history. The window size was set to the number of tokens in the comorbidity description plus two, allowing for tolerance to missing or extra words. During each iteration, the tokens were compared with the comorbidity descriptions using a two-level string-matching algorithm, similar to the process applied for medication matching. To validate the accuracy of this annotation method, a random sample of 80 patient records was manually reviewed by two independent physicians. The overall accuracy was 77% (17% of participants were identified with a false positive medical condition, and 10% with a false negative medical condition).

### Statistical analysis

A descriptive analysis was performed to summarize participant characteristics, with continuous variables presented as means and standard deviations (SDs), and categorical variables as percentages.

The moderation analyses followed a rigorous, five-step approach, as described in detail by Griffin et al. [36], which offers a comprehensive and reader-friendly explanation of this method. First, the sample was stratified by treatment status (statin use versus non-use) and moderator status (concomitant medication use versus non-use). Covariate overlap across the stratified groups was then assessed, and any covariate subgroups showing substantial non-overlap in their ranges (e.g., females in the analysis of 5α-reductase inhibitors) were excluded from further analyses. Second, propensity score (PS) weights were estimated separately within each moderator level to minimize bias, using the TWANG package in R [38, 39]. This method applies generalized boosted modelling—a nonparametric machine-learning approach—to statistically balance exposure groups (statin users vs. non-users) within each level of the moderator on potential confounders. It is important to note, however, that this approach does not balance between the levels of the moderator (e.g., aspirin users vs. non-users). Therefore, while our analysis supports the presence of effect modification, it does not permit causal inference regarding the moderator itself. If covariates contained missing values (NA), TWANG adjusted propensity score weights to also balance missingness rates between treatment and control groups. Third, the effectiveness of PS weights in improving covariate balance within each moderator level was evaluated. Standardized mean differences (SMDs) were used to compare means, while Kolmogorov–Smirnov (KS) statistics were used to assess distributional differences. A threshold of 0.20 was applied for both SMD and KS statistics. Fourth, moderated average treatment effects (M-ATE) were estimated using covariate-adjusted weighted logistic regressions, calculated as the difference in PS-weighted means between statin users and non-users within each moderator group (e.g., beta-blocker users vs. non-users). Average marginal effects (AME) were also calculated within each moderator level to indicate the average change in the outcome due to a one-unit change in the predictor. Additionally, the interaction term between statin use and each moderator (β_3_) was assessed using the following equation: Y = β_0_ + (β_1_ + β_3_M)X + β_2_M; where X represents statin use, M represents the moderator, and Y is the outcome variable. The significance of β_3_, along with its *p*-value, was used to identify statistically significant interactions. Stratified sensitivity analyses were conducted to assess the robustness of findings across subgroups with and without a history of atherosclerotic cardiovascular disease (including stroke, coronary disease, or peripheral artery disease), as well as those with (MMSE ≥ 27) and without (MMSE < 27) normal cognition. Further sensitivity analyses were performed to evaluate the impact of unobserved confounding. For this, the OVtool in R was used to simulate scenarios in which potential unobserved confounders—independent of the observed confounders but conditional on treatment—could influence the estimated treatment effect and its statistical significance (i.e., *p*-value) within each moderator level [40].

All statistical analyses were performed using R version 4.3.3 and RStudio version 2024.04.2, with statistical significance defined as *p* < 0.05 (two-sided). The results should be interpreted with caution because no adjustments were made for multiple hypothesis testing in this exploratory study.

## Results

### Participant characteristics

Among the 8548 first-visit patients at the Basel Mobility Center, 7362 were aged 60 years or older, 424 patients had missing data on gait measurements (usual and/or maximum gait) and 1859 had unknown or incomplete information on medication use. Applying these three exclusion criteria left a total of 5519 patients included in the final analysis. The mean age of the cohort was 76.4 years (SD, 7.4; range 60–100 years), and 54% of the participants were female. Statins were used by 1727 patients (31%). The most common non-statin medications in the cohort were aspirin (28%), followed by β-blockers (25%), sartans (24%), and proton pump inhibitors (20%). Table 1 presents patient demographics before propensity score weighting. Statin users were more likely to have comorbid conditions such as hyperlipidemia, coronary heart disease, vascular disease, hypertension, and diabetes. They were also more likely to use cardiovascular medication such as aspirin, β-blockers, angiotensin-converting enzyme inhibitors (ACE inhibitors), and dihydropyridines as well as antidiabetic medication such as biguanides.

**Table 1:** Demographics of 5519 participants 60 years and older, before propensity score weighting. ACEi = Angiotensin Converting Enzyme inhibitor; SSRI = Selective Serotonin Reuptake Inhibitor

Variable	Overall *N* = 5519	No statin *N* = 3792	Statin *N* = 1727	Standardized mean difference
Age, mean (SD), y	76.43 (7.36)	76.19 (7.55)	76.95 (6.89)	0.11
Sex				0.24
Female	54%	58%	46%	
Male	46%	42%	54%	
Body mass index, mean (SD)	25.73 (4.41)	25.36 (4.29)	26.54 (4.57)	0.27
Physically active	87%	87%	86%	0.04
Walking aid	14%	14%	16%	0.07
Fall in previous year	30%	29%	33%	0.08
Medication				
5α-reductase inhibitor	2.4%	2.1%	3.1%	0.06
ACEi	17%	13%	25%	0.32
Potassium sparing diuretic	2.0%	1.4%	3.4%	0.13
α-adrenergic antagonist	6.7%	5.2%	10%	0.18
Antipsychotic	6.1%	6.4%	5.4%	0.04
Aspirin	28%	19%	48%	0.65
Benzodiazepine	16%	16%	16%	0.01
β-blocker	25%	18%	40%	0.50
Biguanide	9.0%	5.7%	16%	0.34
Dihydropyridine	17%	13%	25%	0.32
Insulin	1.6%	0.9%	3.0%	0.15
Opioid	3.3%	3.4%	3.2%	0.01
Proton pump inhibitor	20%	17%	28%	0.27
Sartan	24%	20%	32%	0.26
SSRI	10%	9.0%	12%	0.11
Loop diuretic	8.5%	6.5%	13%	0.22
Thiazide	14%	12%	17%	0.15
Sulphonylurea	3.2%	2.3%	5.3%	0.15
Tricyclic antidepressant	3.1%	3.3%	2.7%	0.04
Thyroid hormone	5.5%	5.0%	6.7%	0.07
Uric acid inhibitor	3.4%	2.5%	5.4%	0.15
Medical history				
Vascular disease	17%	13%	28%	0.40
Chronic kidney disease	8.8%	7.0%	13%	0.19
Congestive heart failure	18%	15%	25%	0.26
Coronary heart disease	13%	6.3%	27%	0.59
Depression	21%	20%	24%	0.09
Diabetes mellitus	13%	8.9%	21%	0.36
Hip or knee osteoarthritis	24%	24%	24%	0.00
Hyperlipidemia	23%	11%	48%	0.88
Hypertension	48%	43%	61%	0.38
Low back pain	22%	21%	26%	0.13
Parkinson disease	2.4%	2.4%	2.4%	0.00
Pulmonary disease	25%	23%	31%	0.19

### Impact of statin use on GSR

The average GSR for the entire cohort was 41.3 cm/s, while the average usual gait speed was 105 cm/s. Before adjustment, the mean GSR among statin users and non-users was 39.7 cm/s and 42.0 cm/s, respectively (difference of − 2.3 cm/s [95% CI, − 3.7 to − 0.99]). Similarly, the average usual gait speed was 103 cm/s for statin users and 106 cm/s for non-users (difference of − 3.1 cm/s [95% CI, − 4.6 to − 1.6]). After applying propensity score weighting, statin use remained significantly associated with a lower GSR (− 1.9 cm/s [95% CI, − 3.1 to − 0.72]). However, the negative association between statin use and usual gait speed was no longer statistically significant (− 0.90 cm/s, *p* = 0.267).

### Moderating effects of concomitant medication

To evaluate whether concomitant medications moderate the association between statin use and gait speed reserve (GSR), each medication was assessed individually as a potential moderator in separate regression analyses. In the unadjusted moderation models—i.e., without applying the full five-step propensity score (PS) weighting procedure—significant moderating effects were observed for ACE inhibitors (β_3_ = 3.73, *p* = 0.030), aspirin (β_3_ = 6.48, *p* < 0.001), and dihydropyridine calcium channel blockers (β_3_ = 4.07, *p* = 0.019) (Supplementary Table 1). Similarly, significant moderating effects on the association between statins and usual gait speed were found for aspirin (β_3_ = 7.32, *p* < 0.001), dihydropyridine calcium channel blockers (β_3_ = 6.15, *p* = 0.002), beta blockers (β_3_ = 5.20, *p* = 0.002), sulphonamide loop diuretics (β_3_ = 5.78, *p* = 0.024), and proton-pump inhibitors (β_3_ = 3.83, *p* = 0.034) (Supplementary Table 1).

**Fig. 1:**
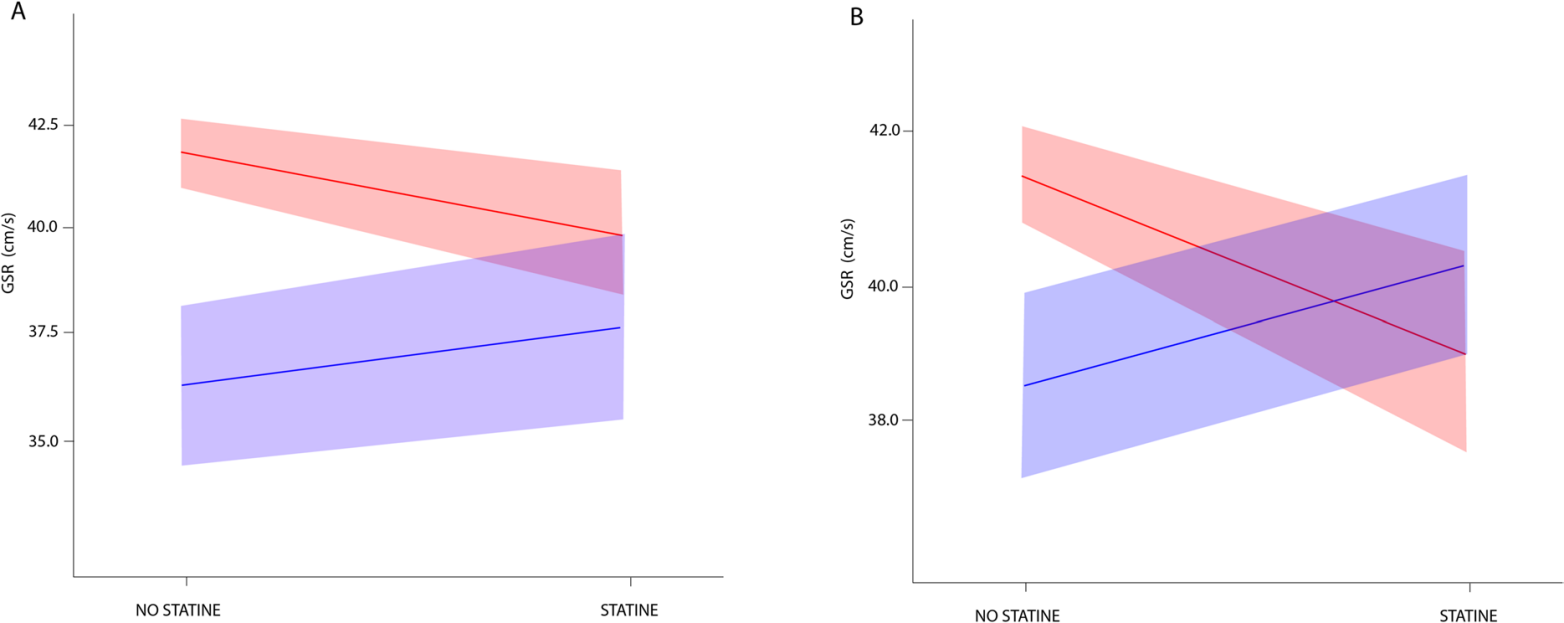
Interaction plots with 95% confidence intervals are presented for ACE inhibitors and statins (**A**) and for aspirin and statins (**B**). The presence of the concomitant medication is represented in blue, while its absence is shown in red

Propensity score–adjusted moderation analyses were then conducted using a rigorous stepwise procedure. Covariate subgroups with substantial non-overlap were excluded from further analyses for 14 of the 21 candidate moderators (Supplementary Table 2). Following propensity score weighting for each potential moderator—where all concomitant medications were included as covariates except the one being examined as a moderator—all standardized mean differences (SMDs) and Kolmogorov–Smirnov (KS) statistics fell below the 0.20 threshold for all covariates (data not shown). There was significant evidence of moderation in the association between statins and GSR for both ACE inhibitors and aspirin, as indicated by the significant interaction terms (β_3_) in the linear regression models: 3.7 [95% CI, 0.0 to 7.4] for ACE inhibitors and 5.8 [95% CI, 2.5 to 9.1] for aspirin (Table 2). The estimated M-ATE (moderated average treatment effect) for statin users compared to non-users in older adults not taking ACE inhibitors was − 2.2 cm/s [95% CI, − 4.0 to − 0.3], suggesting that statins may decrease the physical performance in this group. In contrast, older adults taking ACE inhibitors and statins showed a non-significantly higher GSR (1.5 cm/s [95% CI, − 1.7 to 4.7]) compared to patients taking ACE inhibitors without statins, indicating a potential protective effect of statins on physical performance in those taking ACE inhibitors. Similarly, the estimated M-ATE for statin users versus non-users in older adults not taking aspirin was − 3.4 cm/s [95% CI, − 5.6 to − 1.3], potentially implying that statins may modestly decrease physical performance in this subgroup. However, older adults taking aspirin showed non-significantly higher values in GSR (2.3 cm/s [95% CI, − 0.1 to 4.8]) compared to individuals not taking statins, suggesting that statins may increase physical performance in adults concomitantly taking aspirin. The interactions of statins with ACE inhibitors and aspirin are visualized in the interaction plots (Fig. 1). Post-hoc exploratory analyses of specific statin subtypes in combination with ACE inhibitors or aspirin did not reveal substantial differences among the four most frequently used statins: atorvastatin, simvastatin, rosuvastatin, and pravastatin (Supplementary Table 3).

**Table 2:** Linear regression results for the PS-weighted linear model for outcome gait speed reserve

Moderator	Estimated coefficient	95% CI	*p*-value	M-ATE_0_	M-ATE_1_
5α-reductase inhibitor	5.94	(− 6.58, 18.5)	0.35	− 1.20	4.75
**ACEi**	**3.65**	**(− 0.06, 7.37)**	**0.05**	** − 2.15**	**1.51**
Potassium sparing diuretic	− 2.31	(− 9.75, 5.13)	0.54	− 1.66	− 3.96
α-adrenergic antagonist	5.38	(− 0.14, 10.9)	0.06	− 2.26	3.12
Antipsychotic	2.51	(− 3.96, 8.98)	0.45	− 2.10	0.41
**Aspirin**	**5.76**	**(2.47, 9.05)**	** < 0.001**	** − 3.43**	**2.34**
Benzodiazepine	− 0.44	(− 4.03, 3.15)	0.81	− 1.94	− 2.38
β-blocker	1.70	(− 1.61, 5.01)	0.31	− 2.11	− 0.41
Biguanide	3.48	(− 0.99, 7.94)	0.13	− 2.14	1.34
Dihydropyridine	2.42	(− 1.14, 5.99)	0.18	− 2.30	0.12
Insulin	5.02	(− 4.91, 14.9)	0.32	− 2.12	2.90
Opioid	0.26	(− 6.65, 7.17)	0.94	− 1.89	− 1.63
Proton pump inhibitor	2.09	(− 1.78, 5.96)	0.29	− 2.53	− 0.44
Sartan	0.08	(− 3.41, 3.58)	0.96	− 1.79	− 1.71
SSRI	0.24	(− 4.12, 4.61)	0.91	− 1.99	− 1.75
Loop diuretic	1.49	(− 2.70, 5.69)	0.49	− 2.08	− 0.59
Thiazide	3.48	(− 0.32, 7.28)	0.07	− 2.42	1.06
Sulphonylurea	2.20	(− 4.29, 8.70)	0.51	− 1.85	0.35
Tricyclic antidepressant	− 2.80	(− 12.0, 6.42)	0.55	− 1.81	− 4.61
Thyroid hormone	1.61	(− 4.60, 7.81)	0.61	− 1.92	− 0.32
Uric acid inhibitor	1.90	(− 5.27, 9.07)	0.60	− 1.96	− 0.06

Propensity Score (PS) weighted linear regressions for the secondary outcome usual gait speed are available in Supplementary Table 4. There was no significant evidence of moderation in the association between statins and usual gait speed for any of the concomitant medications.

### Sensitivity analysis

**Fig. 2:**
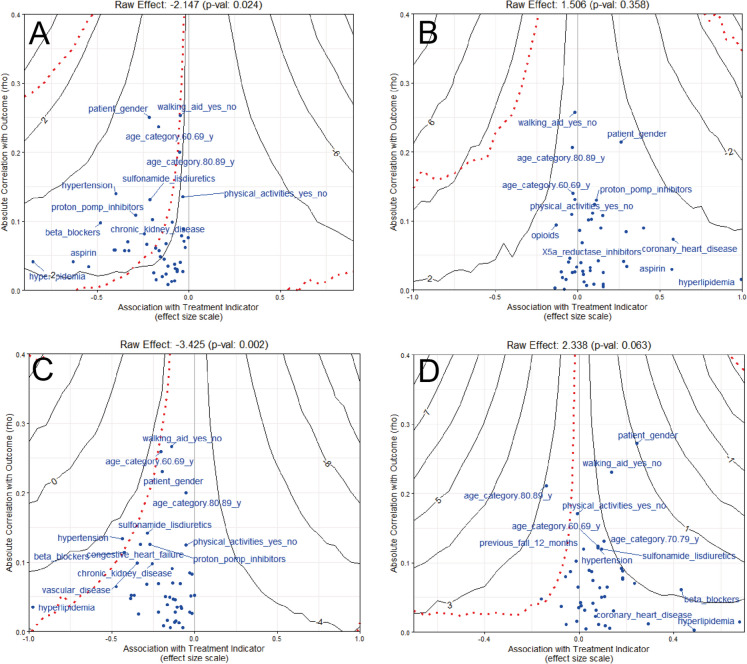
Results from omitted variable sensitivity analyses: **A** no ACEi intake; **B** ACEi intake; **C** no aspirin intake; **D** aspirin intake. Treatment effect contours are indicated in black; the *p*-value threshold line (*p* = 0.05) in red

Stratified sensitivity analyses for primary and secondary atherosclerotic cardiovascular disease prevention reinforced the main findings, demonstrating a positive moderation effect of both ACE inhibitors and aspirin on the relationship between statin use and GSR in both groups. Notably, the effect was strongest in individuals without prior atherosclerotic cardiovascular disease (Supplementary Table 5). Similarly, cognitive stratification did not alter the main conclusions, with significant moderating effects observed in both normal cognition and cognitively impaired subgroups (Supplementary Table 6). Additionally, sensitivity analyses for unobserved confounding were conducted to assess the robustness of the estimated M-ATE effects. Figure 2 illustrates how both the effect size (solid contours in cm/s) and *p*-value (dashed contours) change in response to an unobserved confounder. The confounder’s association with statin use is represented by an effect size or SMD (x-axis), and its relationship with the outcome GSR is expressed as a correlation (y-axis). As the correlation between the unobserved variable and the outcome or treatment group increases, the solid contours reflect how the adjusted M-ATE estimates change. For older adults not taking ACE inhibitors (Fig. 2A), the estimated effect becomes larger as we move right along the x-axis, from − 4 to − 8 cm/s, and crosses the null effect as we move left. The dashed contours show how statistical significance is affected, paralleling the treatment effect contours. Specifically, a confounder with a correlation with GSR lower than 0.10 would need an SMD greater than 0.125 between statin users and non-users to render our results non-significant (*p* < 0.05). Blue dots on the plots show observed correlations and effect sizes for the different categories of the covariates used in the PS weights. Similar plots are shown for older adults taking ACE inhibitors (Fig. 2B), not taking aspirin (Fig. 2C), and taking aspirin (Fig. 2D). Across all plots, observed covariates cluster near the region where unobserved covariates could impact our M-ATE estimates. This suggests that our findings are sensitive to omitted variables, which could shift the results toward the null, and thus should be interpreted with caution.

## Discussion

In this cohort study of 5519 older adults, we found evidence that ACE inhibitors and aspirin moderate the relationship between statin use and gait speed reserve (GSR). Overall, statin use was associated with a slightly reduced gait speed reserve across the full cohort (− 1.9 cm/s, *p* = 0.002). However, in the group taking concomitant ACE inhibitors or aspirin we observed higher gait speed reserves while on statins (1.5 cm/s and 2.3 cm/s, respectively), compared to the group not taking ACE inhibitors or aspirin, who showed lower gait speed reserves while on statins (− 2.2 cm/s and − 3.4 cm/s). We found no statistically significant associations between statin use and usual gait speed—neither an overall association nor any moderating effects of concomitant medications.

To the best of our knowledge, this is the first study to investigate the association between statin use and GSR in older adults. GSR is considered a measure for physiological reserve and a more sensitive indicator of functional change compared to usual gait speed, often used in frailty and intrinsic capacity assessments [15, 16]. The inability to perform beyond baseline physical function, represented by a small GSR, likely reflects a complex interplay of multiple subtle physiological impairments. Muscle strength, pain, proprioception, and vestibular function are examples of physiological factors that can influence GSR [7, 8]. Hypothetically, greater muscle strength and proprioception should result in higher GSR levels, whereas pain, or deficiencies in vestibular function and muscle strength, would lead to reduced GSR levels. However, it should be noted that some studies have questioned the higher reliability of GSR as an indicator of functional outcomes in older adults compared to usual gait speed [41, 42]. Variability in findings may stem from differences in participant characteristics, inclusion criteria and gait assessment methods, as well as the possibility of non-linear relationships between GSR and functional outcomes. For instance, severe cognitively impaired older adults could exhibit a relatively high GSR despite an increased risk of falls. This paradox might occur if, when prompted to walk at maximal speed, they accelerate beyond their “normal” or “safe” maximal speed [43]. GSR and its underlying physiological factors are probably shaped by both pharmacological and non-pharmacological influences such as physical activity, smoking, alcohol consumption, and participation in rehabilitation or lifestyle interventions. Although such non-pharmacological variables have been previously linked to GSR and overall physiological reserve [7, 44], pharmacological influences—especially multi-drug interactions—remain underexplored. Our study addresses this gap but recognizes the need for future work to better integrate both domains.

In our study, the usual gait speeds and GSR values aligned with those reported in other studies of older adults, typically ranging from 70 to 140 cm/s for usual gait speed and 20 to 60 cm/s for GSR [7, 8, 43]. The negative overall association between statin use and GSR in our study, along with the lack of significant association with usual gait speed (albeit small trend towards a negative association, aligning with the idea that static gait performance metrics like usual gait speed are less sensitive for detecting functional changes compared to GSR), is consistent with findings from several large observational studies. For instance, Peeters et al. reported that statin use was linked to poorer physical function (OR 1.29, [99% CI, 1.07–1.55]) in a middle-aged population using the SF-36 physical functioning subscale [45]. Additionally, a longitudinal study of 2500 older patients with peripheral artery disease found no association between statins and the evolution of usual gait speed [46], while Kawai et al., Haerer et al. and Strandberg et al. (Helsinki Businessman Study) reported no significant link between statin use and gait speed (usual or maximum) in cross-sectional studies from Japan (*n* = 1022), Australia (*n* = 500), and Finland (*n* = 1365), respectively [47–50]. Kawai et al., although not significant, observed a lower usual gait speed of 6 cm/s among statin users [47]. Similarly, the Gait and Brain Study, involving 345 older community-dwelling adults, found no significant association with usual gait speed, though a negative trend of − 1.5 cm/s was noted among statin users [51]. Our results are further supported by randomized controlled trials by Parker et al. and Henderson et al. (LIFE Study), which found no significant effect of statins on exercise capacity in middle-aged and older adults [52, 53]. However, our findings contrast with studies suggesting that statins may benefit physical function or protect against accelerated aging. For instance, Dumurgier et al. found a 27% slower decline in usual gait speed among statin users compared to non-users in a 10-year cohort study of 4000 older adults [26]. Similarly, Qazi et al*.* observed a decreased incidence of frailty among statin users in a 10-year cohort study of 1,253,152 older men (HR 0.89, [95% CI, 0.89–0.90]) [54]. Additionally, a meta-analysis by De Vries et al. indicated a protective effect of statins, showing a reduced risk of falls (OR 0.80, [99% CI, 0.65–0.98]) [55]. The variability in findings across these studies may be influenced not only by differences in population characteristics but also by the role of concomitant medications and their interactions with statin use—factors that have not been consistently accounted for. Notably, a recent Chinese study reported that statin users experienced improvements in mobility, including increased usual gait speed and enhanced balance [56]. Interestingly, participants in this study were prescribed thiazide as a concomitant antihypertensive agent. This aligns with our findings in Table 2 and Supplementary Table 4, where older adults on concomitant thiazide exhibited a positive association between statin use and both usual gait speed and GSR.

In our main analyses, ACE inhibitors and aspirin emerged as moderators in the association between statin use and GSR. Specifically, while individuals not taking ACE inhibitors or aspirin showed a reduced ability to perform beyond baseline physical function (i.e., low GSR) when on statins, older adults using statins in combination with ACE inhibitors or aspirin demonstrated a higher GSR. This moderating effect may be partly explained by the shared effects on angiotensin II type 1 receptor (AT1R) signalling within the renin–angiotensin–aldosterone system (RAAS). ACE inhibitors reduce AT1R activation by blocking the conversion of Ang I to Ang II, while statins are known to suppress AT1R activity by downregulating AT1R translation through destabilization of AT1 mRNA [57–59]. Additionally, aspirin has been suggested to inhibit AT1R transcription [60]. Inhibition of RAAS signaling has been shown to provide cardiovascular benefits and potential gero-protective effects [61, 62]. However, other studies suggest that RAAS inhibition may promote features associated with accelerated aging. For instance, Van Ginkel et al. found that ACE inhibition redirects gene activation from muscle fibers to the surrounding interstitium, reducing the transcription of muscular HIF-1α and mitochondrial genes while increasing endothelial VEGF transcription post-exercise. This shift resulted in a lower peak aerobic power ratio between one- and two-legged VO2_max_ tests in the ACEi group compared to controls [63]. In a study involving over 1500 middle-aged participants, Loprinzi et al. reported that ACE inhibitor use was associated with a 37% lower odds (OR = 0.63, 95% CI, 0.48–0.83, *p* = 0.002) of engaging in moderate-to-vigorous physical activity, decreased knee extensor strength (*β* = − 15.4, 95% CI, − 27.2 to − 3.4, *p* = 0.01), and increased time to complete 20-foot (*β* = 0.42, 95% CI, 0.02–0.81, *p* = 0.04) and 8-foot walking tests (*β* = 0.22, 95% CI, 0.05–0.39, *p* = 0.01) [64]. Similarly, Gray et al*.*, in a study of 5777 women aged 65–79, found ACE inhibitor use significantly associated with lower grip strength (22.40 kg vs. 23.18 kg) and a greater decline in 15-s chair-rise performance (− 0.181 vs. − 0.145 chair stands decline annually) [27]. Though RAAS inhibition as a mechanism remains unclear, low-dose aspirin has also been linked to an increased risk of serious falls, as reported in the ASPREE Randomized Clinical Trial (16,703 older adults, median age 74) with 884 falls in the aspirin group versus 804 in the placebo group [65]. Differences in dosages, treatment durations, and study populations may contribute to the observed mixed effects of RAAS inhibition. We speculate that the RAAS inhibition in older adults in our study taking statins, ACE inhibitors, or aspirin could have an overall negative impact on physical function, potentially outweighing benefits through alternative pathways. However, the use of statins with concomitant ACE inhibitors or aspirin may enhance beneficial effects, while the adverse impact of RAAS inhibition might reach a “saturation point,” leading to a net positive effect on physical function compared to the use of ACE inhibitors or aspirin alone.

While the observed effects of ACE inhibitors and aspirin on Gait Speed Reserve (GSR) may appear modest—interaction effects of 3.65 cm/s and 5.76 cm/s, respectively—existing literature suggests that differences of similar magnitudes are clinically meaningful. For instance, Davis et al. reported a GSR difference of just 1.85 cm/s between older adults who later experienced falls and those who did not [8]. Ueno et al. observed an 11% increased mortality risk for every 10 cm/s decrease in GSR among older adults hospitalized for cardiovascular disease [9], and Callisaya et al. identified a significant 4 cm/s difference in GSR between healthy older adults and those with mild cognitive impairment (MCI) [43]. Although a formal minimal clinically important difference (MCID) for GSR has not yet been established—unlike for other functional measures such as grip strength and usual gait speed [66, 67]—these studies suggest that the magnitude of GSR differences observed in our analysis may indeed be clinically relevant.

Our subgroup sensitivity analyses reinforced the moderating effects of ACE inhibitors and aspirin on the statin-GSR association across all subgroups. The strongest effects were observed in individuals without atherosclerotic cardiovascular disease, potentially because statins already provide substantial cardiovascular benefits in affected individuals, leaving less room for additional enhancement of GSR. Similarly, while ACE inhibitors and aspirin showed positive moderating effects in both cognitively intact and impaired participants, the interaction effects were statistically significant and most pronounced in those with normal cognition. The attenuated effects in the cognitively impaired group may be explained by cognitive impairment often reflecting underlying vascular pathology, even in the absence of overt cardiovascular disease, leading to a diminished detrimental effect of statins, reflected in a less negative M-ATE_0_. Regarding ACE inhibitors, sensitivity analyses indicated that our results may be affected by unmeasured confounding, potentially reaching a null effect if unmeasured confounders with correlations similar to those of gender or proton pump inhibitor intake were included. After propensity score weighting, our assessment of standardized mean differences (SMD) indicated a slight remaining imbalance in comorbidities (vascular disease, coronary heart disease, hyperlipidemia) among patients taking ACE inhibitors (SMD between 0.1 and 0.2). Specifically, statin users within this cohort who also take an ACE inhibitor generally had more comorbidities compared to non-statin users on ACE inhibitors. If we were able to balance these differences—resulting in a similar proportion of patients with chronic illnesses in the statin as the non-statin group—we would likely observe an even higher gait speed reserve (GSR) among statin users compared to non-statin users. This implies that the beneficial GSR-effects of statins when used alongside ACE inhibitors could be even more pronounced.

A major strength of this study is its pioneering investigation into the interaction between multiple medications and their effect on functional aging within a large, real-world cohort of older adults. The findings underscore the importance of personalized medication strategies in older adults, particularly for those taking statins. Such strategies should not only address the specific conditions for which medications are prescribed but also consider their broader effects on overall functionality, a critical factor in optimizing quality of life throughout the ageing process. Another notable strength is the use of the accurately measured gait speed reserve (GSR) as an outcome measure. GSR is a dynamic gait metric that serves as an intuitive and sensitive indicator of physiological reserve, making it an early marker for ageing-related clinical outcomes [14, 15]. Integrating GSR monitoring with medication management could facilitate timely interventions to maintain functionality, such as physical therapy or medication adjustments, particularly for patients at risk of functional decline. For statins, this approach balances the cardiovascular benefits against their potential effects on functionality. Additionally, the study accounted for a wide range of covariates, including comorbidities and medication use, by employing propensity score (PS) analysis. This robust methodological framework enhances the reliability and generalizability of the findings. However, there are also some limitations that warrant consideration. Most notably, the cross-sectional and observational nature of this study limits our ability to infer causal relationships or determine the temporal sequence between drug use and GSR. While we adjusted for numerous potential confounders, provided biologically plausible interpretations, and conducted extensive sensitivity analyses, reverse causation remains a plausible explanation. For example, physicians may tailor prescribing practices based on patients’ physical functioning, although our stratified results suggest more complex patterns. Future longitudinal studies—and ideally, randomized controlled trials—are needed to clarify temporal ordering and address potential residual confounding. Particular attention should be given to unmeasured factors such as nutritional status, physical activity (objectively measured), comorbidity severity and progression, and lifestyle factors (e.g., smoking and alcohol use), all of which may influence the observed associations. Additionally, while we discuss potential biological mechanisms underlying the observed interactions—such as modulation of RAAS signaling—these remain hypothetical and were not directly proven in this study. Experimental and longitudinal research is needed to validate these mechanistic hypotheses and further explore the biological causal pathways involved. Second, the study did not formally account for dosage, treatment duration, or specific subtypes of statins (lipophilic vs. hydrophilic), which may influence the results and could affect patient’s response [23, 57]. Importantly, future studies addressing these factors will require sufficiently large sample sizes for each statin subtype, as dosage comparisons across different statins are not straightforward. Cholesterol-lowering equivalence doses do not necessarily reflect equivalent functional effects of statins, making direct dose–response comparisons challenging. Third, since this study was conducted at a single geriatric mobility center, there is a risk of selection bias that may affect the generalizability of the findings. Patients referred to such centers are typically evaluated for mobility concerns and may differ from the broader older adult population in terms of baseline functional status, comorbidity burden, and medication profiles. In addition, although we did not identify systematic reasons for missing data, its presence introduces an inherent risk of selection bias. To move from a proof-of-principle to a more generalizable proof-of-concept, future studies should aim to replicate these findings across different clinical settings and geographic regions. Fourth, we did not apply corrections for multiple statistical testing, as this exploratory study prioritized minimizing false negatives over avoiding false positives. This decision reflects the study’s goal of generating hypotheses for future research rather than drawing definitive conclusions. Fifth, while we recorded prescribed and reported medication use, we could not verify whether participants consistently took their medications as prescribed. Variability in adherence—coupled with an estimated medication annotation accuracy of 93%—may have introduced misclassification bias, potentially influencing the observed associations between statin use, concomitant medications, and GSR. Future studies should incorporate objective adherence measures, such as pharmacy refill records or biochemical verification, to better assess the impact of medication adherence on mobility outcomes. Finally, our analysis was limited to the interaction between two drugs at a time, without considering higher-order drug interactions. While this approach allowed us to identify potential moderation effects, it does not fully capture the complexity of polypharmacy in older adults, where multiple medications may interact simultaneously. Future large sample-size studies should explore machine learning and network-based approaches to investigate complex medication interactions, which could provide a more comprehensive understanding of polypharmacy effects on physiological reserve and mobility.

## Conclusions

Our findings suggest that combining statins with ACE inhibitors or aspirin can be beneficial for improving physiological reserves as measured by GSR. These results highlight the importance of considering concomitant medication use when evaluating the effects of statins on functional outcomes in aging populations. While this study offers novel insights into the interaction between statins and commonly prescribed medications, its observational nature limits causal inference. Future research should prioritize interventional designs—particularly randomized controlled trials—to confirm these findings and explore the underlying biological mechanisms. In parallel, longitudinal studies and advanced analytical approaches, such as machine learning, may help uncover complex multi-drug interactions and refine our understanding of their impact on physiological reserve in aging populations.

## Supplementary Information

Below is the link to the electronic supplementary material.

Supplementary file1 (DOCX 23 KB)

## Data Availability

The pseudonymized participant data that support the findings of this study are available from the corresponding author upon reasonable request.

## References

[1] WHO. Ageing and health. Accessed October 26th, 2024. https://www.who.int/health-topics/ageing#tab=tab_1

[2] Calvani R, Marini F, Cesari M, et al. Biomarkers for physical frailty and sarcopenia: state of the science and future developments. Review Journal of Cachexia Sarcopenia and Muscle. 2015;6(4):278–86. 10.1002/jcsm.12051. 26675566 10.1002/jcsm.12051 PMC4670735

[3] Chhetri J, Xue Q, Ma L, Chan P, Varadhan R. Intrinsic capacity as a determinant of physical resilience in older adults. J nutri health & aging. 2021;25(8):1006–11. 10.1007/s12603-021-1629-z. 10.1007/s12603-021-1629-z PMC8035602 34545921

[4] Whitson H, Cohen H, Schmader K, Morey M, Kuchel G, Colon-Emeric C. Physical resilience: not simply the opposite of frailty. J American Geriatrics Soc. 2018;66(8):1459–61. 10.1111/jgs.15233. 10.1111/jgs.15233 PMC6157007 29577234

[5] Partridge L, Fuentealba M, Kennedy BK. The quest to slow ageing through drug discovery. Nat Rev Drug Discov. 2020;19(8):513–32. 10.1038/s41573-020-0067-7. 32467649 10.1038/s41573-020-0067-7

[6] López-Otín C, Blasco MA, Partridge L, Serrano M, Kroemer G. Hallmarks of aging: AN expanding universe. Cell. 2023;186(2):243–78. 10.1016/j.cell.2022.11.001. 36599349 10.1016/j.cell.2022.11.001

[7] Lindholm B, Basna R, Ekström H, Elmståhl S, Siennicki-Lantz A. Gait speed reserve in the general population-based ‘Good Aging in Skåne’ cohort study-distribution and associated factors. Geroscience. 2024. 10.1007/s11357-024-01318-6. 39192005 10.1007/s11357-024-01318-6 PMC11872813

[8] Davis JRC, Knight SP, Donoghue OA, et al. Comparison of gait speed reserve, usual gait speed, and maximum gait speed of adults aged 50+ in Ireland using explainable machine learning. Front Netw Physiol. 2021;1: 754477. 10.3389/fnetp.2021.754477. 36925580 10.3389/fnetp.2021.754477 PMC10013005

[9] Ueno K, Kamiya K, Hamazaki N, et al. Usefulness of measuring maximal gait speed in conjunction with usual gait speed for risk stratification in patients with cardiovascular disease. Exp Gerontol. 2022;164: 111810. 10.1016/j.exger.2022.111810. 35452782 10.1016/j.exger.2022.111810

[10] Yoshikoshi S, Yamamoto S, Suzuki Y, et al. Reserved gait capacity and mortality among patients undergoing hemodialysis. Nephrol Dial Transplant. 2023;38(12):2704–12. 10.1093/ndt/gfad109. 37259268 10.1093/ndt/gfad109

[11] García A, Ródenas I, Molina R, et al. Gait plasticity impairment as an early frailty biomarker. Exper Gerontol. 2020;142:111137. 10.1016/j.exger.2020.111137. 33122128 10.1016/j.exger.2020.111137

[12] do Carmo Correia de Lima M, Loffredo Bilton T, Jefferson de Sousa Soares W, Paccini Lustosa L, Ferriolli E, Rodrigues PM. Maximum walking speed can improve the diagnostic value of frailty among community-dwelling older adults a cross-sectional study. The J Frailty Aging 2019;8(1):39–41 10.14283/jfa.2018.44 10.14283/jfa.2018.44 PMC12275780 30734830

[13] Hirai T, Kamide N, Shigeta K. Decrease in maximum paced walking speed predicts hospitalization in community-dwelling older people with disabilities. Eur Geriatr Med. 2023;14(5):961–8. 10.1007/s41999-023-00801-1. 37249736 10.1007/s41999-023-00801-1

[14] Yoshikoshi S, Yamamoto S, Suzuki Y, et al. Reserved gait capacity and mortality among patients undergoing hemodialysis. Nephrol Dial Transplant. 2023;38(12):2704–12. 10.1093/ndt/gfad109. 37259268 10.1093/ndt/gfad109

[15] Noguerón García A, Huedo Ródenas I, García Molina R, et al. Gait plasticity impairment as an early frailty biomarker. Exp Gerontol. 2020;142: 111137. 10.1016/j.exger.2020.111137. 33122128 10.1016/j.exger.2020.111137

[16] do Carmo Correia de Lima M, Loffredo Bilton T, Jefferson de Sousa Soares W, Paccini Lustosa L, Ferriolli E, Rodrigues PM. Maximum walking speed can improve the diagnostic value of frailty among community-dwelling older adults a cross-sectional study. J Frailty Aging. 2019;8(1):39–41. 10.14283/jfa.2018.44 10.14283/jfa.2018.44 PMC12275780 30734830

[17] Mellen RH, Girotto OS, Marques EB, et al. Insights into pathogenesis, nutritional and drug approach in sarcopenia: a systematic review. *Biomedicines*. 2023;11(1)10.3390/biomedicines11010136 10.3390/biomedicines11010136 PMC9856128 36672642

[18] Sonaye HV, Sheikh RY, Doifode CA. Drug repurposing: iron in the fire for older drugs. Biomed Pharmacother. 2021;141: 111638. 10.1016/j.biopha.2021.111638. 34153846 10.1016/j.biopha.2021.111638

[19] Davignon J. Beneficial cardiovascular pleiotropic effects of statins. *Circulation*. 2004;109(23 Suppl 1):Iii39–43. 10.1161/01.CIR.0000131517.20177.5a 10.1161/01.CIR.0000131517.20177.5a 15198965

[20] Shishehbor MH, Brennan ML, Aviles RJ, et al. Statins promote potent systemic antioxidant effects through specific inflammatory pathways. Circulation. 2003;108(4):426–31. 10.1161/01.Cir.0000080895.05158.8b. 12860913 10.1161/01.CIR.0000080895.05158.8B

[21] Krysiak R, Okopień B, Herman Z. Effects of HMG-CoA reductase inhibitors on coagulation and fibrinolysis processes. Drugs. 2003;63(17):1821–54. 10.2165/00003495-200363170-00005. 12921488 10.2165/00003495-200363170-00005

[22] Morofuji Y, Nakagawa S, Ujifuku K, et al. Beyond lipid-lowering: effects of statins on cardiovascular and cerebrovascular diseases and cancer. *Pharmaceuticals (Basel)*. 2022;15(2)10.3390/ph15020151 10.3390/ph15020151 PMC8877351 35215263

[23] De Spiegeleer A KH, Crombez L, Descamps A, Rössler R, Kressig RW, et al. Potential role of statins in treatment of acute sarcopenia. *Med Hypotheses*. 2023;

[24] Ridker PM, Danielson E, Fonseca FA, et al. Rosuvastatin to prevent vascular events in men and women with elevated C-reactive protein. N Engl J Med. 2008;359(21):2195–207. 10.1056/NEJMoa0807646. 18997196 10.1056/NEJMoa0807646

[25] Grinspoon SK, Fitch KV, Zanni MV, et al. Pitavastatin to prevent cardiovascular disease in HIV Infection. N Engl J Med. 2023;389(8):687–99. 10.1056/NEJMoa2304146. 37486775 10.1056/NEJMoa2304146 PMC10564556

[26] Dumurgier J, Singh-Manoux A, Tavernier B, Tzourio C, Elbaz A. Lipid-lowering drugs associated with slower motor decline in the elderly adults. J Gerontol A Biol Sci Med Sci. 2014;69(2):199–206. 10.1093/gerona/glt140. 24097424 10.1093/gerona/glt140 PMC5382229

[27] Gray SL, Aragaki AK, LaMonte MJ, et al. Statins, angiotensin-converting enzyme inhibitors, and physical performance in older women. J Am Geriatr Soc. 2012;60(12):2206–14. 10.1111/jgs.12029. 23176078 10.1111/jgs.12029 PMC3521070

[28] Powell M, Koenecke A, Byrd JB, et al. Ten rules for conducting retrospective pharmacoepidemiological analyses: example COVID-19 study. Front Pharmacol. 2021;12: 700776. 10.3389/fphar.2021.700776. 34393782 10.3389/fphar.2021.700776 PMC8357144

[29] Schnegg D, Senn N, Bugnon O, Schwarz J, Mueller Y. Drug prescription in older Swiss men and women followed in family medicine. Drugs-real world outcomes. 2020;7(1):87–95. 10.1007/s40801-019-00175-6. 31845213 10.1007/s40801-019-00175-6 PMC7060976

[30] Kardas P, Lichwierowicz A, Urbanski F, Chudzynska E, Czech M, Kardas G. Prevalence of chronic polypharmacy in community-dwelling elderly people in Poland: analysis of national real-world database helps to identify high risk group. Front In Pharmacol. 2021;12:739740. 10.3389/fphar.2021.739740. 10.3389/fphar.2021.739740 PMC8637161 34867347

[31] GIP ZN. Top 10: Most Commonly used drug classes by polypharmacy patients aged 65 and over in 2023. 2024;

[32] Bronselaer A, De Tré G. A possibilistic approach to string comparison. IEEE Trans Fuzzy Syst. 2009;17(1):208–23. 10.1109/TFUZZ.2008.2008025.

[33] Bronselaer A, De Tré G. Properties of possibilistic string comparison. iEEE Transac on Fuzzy Systems. 2010;18(2):312–325. 10.1109/TFUZZ.2010.2041353

[34] Zhao C, Sahni S. String correction using the Damerau-Levenshtein distance. *BMC Bioinformatics*. 2019;20(Suppl 11):277. 10.1186/s12859-019-2819-0 10.1186/s12859-019-2819-0 PMC6551241 31167641

[35] Kressig RW, Beauchet O. Guidelines for clinical applications of spatio-temporal gait analysis in older adults. Aging Clin Exp Res. 2006;18(2):174–6. 10.1007/bf03327437. 16702791 10.1007/BF03327437

[36] Griffin B, Schuler M, Cefalu M, et al. A tutorial for propensity score weighting for moderation analysis with categorical variables. Med Care. 2023;61(12):836–45. 10.1097/MLR.0000000000001922. 37782463 10.1097/MLR.0000000000001922 PMC10840831

[37] Griffin BA, Schuler MS, Cefalu M, et al. A tutorial for propensity score weighting for moderation analysis with categorical variables: an application examining smoking disparities among sexual minority adults. Med Care. 2023;61(12):836–45. 10.1097/mlr.0000000000001922. 37782463 10.1097/MLR.0000000000001922 PMC10840831

[38] Green KM, Stuart EA. Examining moderation analyses in propensity score methods: application to depression and substance use. J Consult Clin Psychol. 2014;82(5):773–83. 10.1037/a0036515. 24731233 10.1037/a0036515 PMC4172552

[39] McCaffrey DF, Ridgeway G, Morral AR. Propensity score estimation with boosted regression for evaluating causal effects in observational studies. Psychol Methods. 2004;9(4):403–25. 10.1037/1082-989x.9.4.403. 15598095 10.1037/1082-989X.9.4.403

[40] Pane J, Griffin BA, Burgette L, McCaffrey D. OVtool - omitted variable tool. *The Comprehensive R Archive Network*. 2021;

[41] Middleton A, Fulk G, Herter T, Beets M, Donley J, Fritz S. Self-selected and maximal walking speeds provide greater insight into fall status than walking speed reserve among community-dwelling older adults. American J Physic Med & Rehab. 2016;95:475–82. 10.1097/PHM.0000000000000488. 10.1097/PHM.0000000000000488 PMC4912425 27003205

[42] Gregg E, Beggs C, Bissas A, Nicholson G. A machine learning approach to identify important variables for distinguishing between fallers and non-fallers in older women. Plos One. 2023;18:e0293729. 10.1371/journal.pone.0293729. 37906588 10.1371/journal.pone.0293729 PMC10617741

[43] Callisaya ML, Launay CP, Srikanth VK, Verghese J, Allali G, Beauchet O. Cognitive status, fast walking speed and walking speed reserve-the Gait and Alzheimer Interactions Tracking (GAIT) study. Geroscience. 2017;39(2):231–9. 10.1007/s11357-017-9973-y. 28374167 10.1007/s11357-017-9973-y PMC5411364

[44] Prommaban A, Moonkayaow S, Phinyo P, Siviroj P, Sirikul W, Lerttrakarnnon P. The effect of exercise program interventions on frailty, clinical outcomes, and biomarkers in older adults: a systematic review. J Clinic Med. 2024;13(21):6570. 10.3390/jcm13216570. 10.3390/jcm13216570 PMC11547147 39518709

[45] Peeters G, Tett SE, Conaghan PG, Mishra GD, Dobson AJ. Is statin use associated with new joint-related symptoms, physical function, and quality of life? Results from two population-based cohorts of women. Arthritis Care Res (Hoboken). 2015;67(1):13–20. 10.1002/acr.22389. 24964875 10.1002/acr.22389

[46] Lo-Ciganic WH, Perera S, Gray SL, et al. Statin use and decline in gait speed in community-dwelling older adults. J Am Geriatr Soc. 2015;63(1):124–9. 10.1111/jgs.13134. 25537649 10.1111/jgs.13134 PMC4300263

[47] Kawai H, Ihara K, Kera T, et al. Association between statin use and physical function among community-dwelling older Japanese adults. Geriatr Gerontol Int. 2018;18(4):623–30. 10.1111/ggi.13228. 29278297 10.1111/ggi.13228

[48] Haerer W, Delbaere K, Bartlett H, Lord SR, Rowland J. Relationships between HMG-CoA reductase inhibitors (statin) use and strength, balance and falls in older people. Intern Med J. 2012;42(12):1329–34. 10.1111/j.1445-5994.2011.02622.x. 22032261 10.1111/j.1445-5994.2011.02622.x

[49] Strandberg T, Urtamo A, Kähärä J, Strandberg A, Pitkälä K, Kautiainen H. Statin Treatment is associated with a neutral effect on health-related quality of life among community-dwelling octogenarian men: the Helsinki businessmen study. J Gerontol Series a-Biol Sci Med Sci. 2018;73(10):1418–23. 10.1093/gerona/gly073. 10.1093/gerona/gly073 29659717

[50] Strandberg T, Lindström L, Jyväkorpi S, Urtamo A, Pitkälä K, Kivimäki M. Phenotypic frailty and multimorbidity are independent 18-year mortality risk indicators in older men: the Helsinki businessmen study (HBS). Euro Geriat Med. 2021;12(5):953–61. 10.1007/s41999-021-00472-w. 10.1007/s41999-021-00472-w PMC8463371 33661507

[51] Osman A, Speechley M, Ali S, Montero-Odasso M. Fall-risk-increasing drugs and gait performance in community-dwelling older adults: exploratory results from the gait and brain study. Drugs Aging. 2023;40(8):721–30. 10.1007/s40266-023-01045-1. 37347412 10.1007/s40266-023-01045-1

[52] Parker BA, Capizzi JA, Grimaldi AS, et al. Effect of statins on skeletal muscle function. Circulation. 2013;127(1):96–103. 10.1161/circulationaha.112.136101. 23183941 10.1161/CIRCULATIONAHA.112.136101 PMC4450764

[53] Henderson RM, Lovato L, Miller ME, et al. Effect of statin use on mobility disability and its prevention in at-risk older adults: the LIFE study. J Gerontol A Biol Sci Med Sci. 2016;71(11):1519–24. 10.1093/gerona/glw057. 26988662 10.1093/gerona/glw057 PMC5055646

[54] Qazi S, Farah M, Charest B, et al. New statin use is associated with a lower risk of incident frailty in US veterans aged 65 years and older. American J Preventive Cardiol. 2023;15:100540. 10.1016/j.ajpc.2023.100540.

[55] de Vries M, Seppala LJ, Daams JG, van de Glind EMM, Masud T, van der Velde N. Fall-Risk-increasing drugs: a systematic review and meta-analysis: I. Cardiovascular Drugs. *J Am Med Dir Assoc*. 2018;19(4):371.e1–371.e9. 10.1016/j.jamda.2017.12.013 10.1016/j.jamda.2017.12.013 29396189

[56] Ge J, Qin X, Yu X, et al. Amelioration of gait and balance disorders by rosuvastatin is associated with changes in cerebrovascular reactivity in older patients with hypertensive treatment. Hypertension Res 2024;10.1038/s41440-024-01720-9 10.1038/s41440-024-01720-9 38769134

[57] Endres M, Laufs U. Effects of statins on endothelium and signaling mechanisms. Stroke. 2004;35(11 Suppl 1):2708–11. 10.1161/01.Str.0000143319.73503.38. 15375300 10.1161/01.STR.0000143319.73503.38

[58] Kingsley J, Torimoto K, Hashimoto T, Eguchi S. Angiotensin II inhibition: a potential treatment to slow the progression of sarcopenia. Clin Sci (Lond). 2021;135(21):2503–20. 10.1042/cs20210719. 34751393 10.1042/CS20210719

[59] Bueno V, Frasca D. Mini-review: Angiotensin- converting enzyme 1 (ACE1) and the impact for diseases such as Alzheimer’s disease, sarcopenia, cancer, and COVID-19. Front Aging. 2023;4:1117502. 10.3389/fragi.2023.1117502. 36756193 10.3389/fragi.2023.1117502 PMC9899811

[60] Mitra S, Wang X, Khaidakov M, et al. Aspirin downregulates angiotensin type 1 receptor transcription implications in capillary formation from endothelial cells. J Cardiovasc Pharmacol. 2012;60(2):187–92. 10.1097/FJC.0b013e31825b61e2. 22561363 10.1097/FJC.0b013e31825b61e2

[61] Gouveia F, Camins A, Ettcheto M, et al. Targeting brain renin-angiotensin system for the prevention and treatment of Alzheimer’s disease: past, present and future. Ageing Res Rev. 2022;77: 101612. 10.1016/j.arr.2022.101612. 35346852 10.1016/j.arr.2022.101612

[62] De Spiegeleer A, Bronselaer A, Teo JT, et al. The effects of ARBs, ACEis, and statins on clinical outcomes of COVID-19 infection among nursing home residents. J Am Med Dir Assoc. 2020;21(7):909-914.e2. 10.1016/j.jamda.2020.06.018. 32674818 10.1016/j.jamda.2020.06.018 PMC7294267

[63] van Ginkel S, Ruoss S, Valdivieso P, et al. ACE inhibition modifies exercise-induced pro-angiogenic and mitochondrial gene transcript expression. Scand J Med Sci Sports. 2016;26(10):1180–7. 10.1111/sms.12572. 26407530 10.1111/sms.12572

[64] Loprinzi PD, Loenneke JP. The effects of antihypertensive medications on physical function. Prev Med Rep. 2016;3:264–9. 10.1016/j.pmedr.2016.03.009. 27419024 10.1016/j.pmedr.2016.03.009 PMC4929186

[65] Barker AL, Morello R, Thao LTP, et al. Daily low-dose aspirin and risk of serious falls and fractures in healthy older people: a substudy of the ASPREE randomized clinical trial. JAMA Intern Med. 2022;182(12):1289–97. 10.1001/jamainternmed.2022.5028. 36342703 10.1001/jamainternmed.2022.5028 PMC9641595

[66] Bohannon RW, Glenney SS. Minimal clinically important difference for change in comfortable gait speed of adults with pathology: a systematic review. J Eval Clin Pract. 2014;20(4):295–300. 10.1111/jep.12158. 24798823 10.1111/jep.12158

[67] Bohannon RW. Minimal clinically important difference for grip strength: a systematic review. J Physic Therapy Sci. 2019;31(1):75–8. 10.1589/jpts.31.75. 10.1589/jpts.31.75 PMC6348186 30774209

