# Supplementary material for: The association between statins and gait speed reserve in older adults: effects of concomitant medication
Source: GeroScience. 2025 May 7;48(1):777–92. doi: 10.1007/s11357-025-01682-x (PMC12972418; doi:10.1007/s11357-025-01682-x)
Supplement: Supplementary file 1 — Supplementary file1 (DOCX 23 KB) [file 11357_2025_1682_MOESM1_ESM.docx]

**Supplementary Information**

Supplementary Table 1. Linear regression results for the unadjusted linear model for outcome Gait Speed Reserve and Usual Gait Speed.

|  | **Gait Speed Reserve** | | **Usual Gait Speed** | |
| --- | --- | --- | --- | --- |
| **Moderator** | **Estimated Coefficient** | **P-value** | **Estimated Coefficient** | **P-value** |
| 5α-reductase inhibitor | -1.619 | 0.699 | -3.648 | 0.440 |
| ACE inhibitor | 3.733 | 0.030 | 2.337 | 0.229 |
| Aldosterone antagonist and other potassium-sparing agent | 1.400 | 0.757 | 1.836 | 0.719 |
| Alpha-adrenergic antagonist | 2.044 | 0.422 | 0.063 | 0.983 |
| Antipsychotic | 0.226 | 0.938 | 0.344 | 0.917 |
| Aspirin | 6.476 | <0.001 | 7.321 | <0.001 |
| Benzodiazepine | -0.901 | 0.626 | -3.036 | 0.146 |
| Beta-blocker | 2.033 | 0.180 | 5.197 | 0.002 |
| Biguanide | 2.381 | 0.290 | 1.338 | 0.598 |
| Dihydropyridine | 4.067 | 0.019 | 6.147 | 0.002 |
| Insulin or analogue | 3.294 | 0.522 | -2.181 | 0.707 |
| Opioid | -0.207 | 0.957 | -3.190 | 0.460 |
| Proton-pump inhibitor | 1.696 | 0.291 | 3.825 | 0.034 |
| Sartan | -0.870 | 0.572 | 0.502 | 0.773 |
| SSRI | -0.523 | 0.810 | -0.211 | 0.931 |
| Sulfonamide lisdiuretic | 2.161 | 0.341 | 5.782 | 0.024 |
| Sulfonamide thiazide-like | 2.409 | 0.203 | 3.244 | 0.129 |
| Sulfonylurea | 4.062 | 0.257 | 6.714 | 0.097 |
| Tricyclic antidepressant | 0.211 | 0.959 | -0.301 | 0.948 |
| Thyroid hormone | -0.058 | 0.984 | 4.294 | 0.184 |
| Uric acid inhibitor | 0.563 | 0.872 | -0.353 | 0.929 |

Supplementary Table 2. Excluded subgroups and final included amount of adults in propensity score weighting analysis per moderator

| **Moderator** | **Excluded from analysis** | **N included after overlap correction (%)** |
| --- | --- | --- |
| 5α-reductase inhibitor | Females  Underweight + Obesity class II + Obesity class III + BMI Unknown (N/A)  TCA + opioids | 2 429 (40.0%) |
| ACE inhibitor | Walking aid Unknown | 5 497 (99.6%) |
| Aldosterone antagonist and other potassium-sparing agent | 90-99 y  Obesity class III  Parkinson disease  TCA + insulins or analogues | 5 387 (97.6%) |
| α-adrenergic antagonist | Underweight + Obesity class III | 5 353 (97.0%) |
| Antipsychotic | Underweight  Walking aid Unknown (N/A)  Physical activity Unknown (N/A) | 5 202 (94.3%) |
| Aspirin | - | 5 519 (100%) |
| Benzodiazepine | - | 5 519 (100%) |
| Beta-blocker | - | 5 519 (100%) |
| Biguanide | - | 5 519 (100%) |
| Dihydropyridine | BMI Unknown (N/A) | 5 498 (99.6%) |
| Insulin or analogue | Underweight + Obesity class III + BMI Unknown (N/A)  Walking aid Unknown (N/A)  Physical activity Unknown (N/A)  Aldosterone antagonist and other potassium-sparing agent | 5 159 (93.5%) |
| Opioid | 5α-reductase inhibitor | 5 519 (100%) |
| Proton-pump inhibitor | - | 5 519 (100%) |
| Sartan | - | 5 519 (100%) |
| SSRI | BMI Unknown (N/A)  Walking aid Unknown (N/A) | 5 485 (99.4%) |
| Sulphonamide loop diuretic | Obesity class III + BMI Unknown (N/A)  Walking aid Unknown (N/A) | 5 452 (98.8%) |
| Sulphonamide thiazide-like | BMI Unknown (N/A)  Walking aid Unknown (N/A) | 5 485 (99.4%) |
| Sulphonylurea | 90-99 y  Underweight + Obesity class III  Walking aid Unknown (N/A)  Parkinson disease  5α-reductase inhibitor | 5 235 (94.9%) |
| Tricyclic antidepressant | 90-99 y  Obesity class III + BMI Unknown (N/A)  Physical activity Unknown (N/A)  5α-reductase inhibitor + uric acid inhibitor + aldosterone antagonist and other potassium-sparing agent | 5 203 (94.3%) |
| Thyroid hormone | 90-99 y  Obesity class III + BMI Unknown (N/A) | 5 367 (97.2%) |
| Uric acid inhibitor | Underweight + Obesity class III  TCA | 5 353 (97.0%) |

Supplementary Table 3. Linear regression results for the PS-weighted linear model for outcome Gait Speed Reserve for the individual statins.

| **Moderator** | **Estimated Coefficient** | **95% CI** | **P-value** | **M-ATE_0_** | **M-ATE_1_** |
| --- | --- | --- | --- | --- | --- |
| **Simvastatin (n=374)** |  |  |  |  |  |
| ACEi | 2.10 | (-6.09, 10.3) | 0.62 | -4.32 | -2.22 |
| Aspirin | 8.93 | (2.74, 15.1) | 0.005 | -6.08 | 2.86 |
| **Pravastatin (n=192)** |  |  |  |  |  |
| ACEi | 6.64 | (-1.55, 14.8) | 0.11 | -4.49 | 2.15 |
| Aspirin | 6.71 | (-1.43, 14.8) | 0.11 | -5.19 | 1.52 |
| **Fluvastatin (n=28)** |  |  |  |  |  |
| ACEi | -1.97 | (-19.4, 15.5) | 0.83 | -0.44 | -2.40 |
| Aspirin | -6.99 | (-29.8, 15.9) | 0.55 | 0.19 | -6.81 |
| **Atorvastatin (n=885)** |  |  |  |  |  |
| ACEi | 4.15 | (-0.96, 9.25) | 0.11 | -1.27 | 2.88 |
| Aspirin | 5.09 | (0.50, 9.68) | 0.03 | -2.31 | 2.78 |
| **Rosuvastatin (n=239)** |  |  |  |  |  |
| ACEi | 2.90 | (-6.44, 12.2) | 0.54 | -2.07 | 0.83 |
| Aspirin | 4.71 | (-2.91, 12.3) | 0.23 | -2.63 | 2.08 |
| **Pitavastatin (n=9)**  **Iovastatin (n=0)**  **Cerivastatin (n=0)** |  |  |  |  |  |

Supplementary Table 4. Linear regression results for the PS-weighted linear model for outcome Usual Gait Speed.

|  | **Usual Gait Speed** | | | | | |
| --- | --- | --- | --- | --- | --- | --- |
| **Moderator** | **Estimated Coefficient** | **Standard Error** | **P-value** | **95% CI** | **AME (0)** | **AME (1)** |
| 5α-reductase inhibitor | -5.85 | 4.88 | 0.23 | (-15.4, 3.72) | 0.86 | -4.99 |
| ACE-inhibitor | -1.53 | 2.59 | 0.56 | (-6.60, 3.54) | -0.19 | -1.72 |
| Aldosterone antagonist and other potassium-sparing agent | -3.52 | 4.74 | 0.46 | (-12.8, 5.78) | -0.76 | -4.28 |
| α-adrenergic antagonist | -0.03 | 3.58 | 0.99 | (-7.05, 6.99) | -1.10 | -1.13 |
| Antipsychotic | -0.06 | 3.64 | 0.99 | (-7.08, 7.21) | -1.43 | -1.36 |
| Aspirin | 0.54 | 2.24 | 0.81 | (-3.84, 4.93) | -0.49 | 0.05 |
| Benzodiazepine | -0.99 | 3.07 | 0.75 | (-7.01, 5.03) | -1.06 | -2.05 |
| Beta-blocker | 2.45 | 2.33 | 0.29 | (-2.12, 7.02) | -1.16 | 1.29 |
| Biguanide | 0.33 | 2.88 | 0.91 | (-5.32, 5.97) | -0.57 | -0.24 |
| Dihydropyridine | 1.89 | 2.43 | 0.44 | (-2.87, 6.65) | -1.17 | 0.72 |
| Insulin or analogue | -5.89 | 5.55 | 0.29 | (-16.8, 4.99) | -1.23 | -7.13 |
| Opioid Use | -4.83 | 5.02 | 0.34 | (-14.7, 5.01) | -0.81 | -5.64 |
| Proton-pump inhibitor | 0.95 | 2.46 | 0.70 | (-3.87, 5.76) | -0.85 | 0.10 |
| Sartan | -1.36 | 2.30 | 0.56 | (-5.86, 3.15) | -0.62 | -1.98 |
| SSRI | -2.51 | 3.03 | 0.41 | (-8.44, 3.42) | -0.31 | -2.82 |
| Sulphonamide loop diuretic | 2.43 | 2.79 | 0.38 | (-3.03, 7.90) | -1.13 | 1.31 |
| Sulphonamide thiazide-like | 1.27 | 2.63 | 0.63 | (-3.90, 6.43) | -0.62 | 0.65 |
| Sulphonylurea | 3.36 | 4.80 | 0.48 | (-6.05, 12.8) | -1.02 | 2.34 |
| Tricyclic antidepressant | -1.31 | 3.84 | 0.73 | (-8.85, 6.22) | -1.11 | -2.43 |
| Thyroid hormone | 3.05 | 3.71 | 0.41 | (-4.22, 10.3) | -1.00 | 2.04 |
| Uric acid inhibitor | 0.30 | 4.31 | 0.95 | (-8.15, 8.75) | -1.31 | -1.01 |

Supplementary Table 5. Linear regression results for the PS-weighted linear model for outcome Gait Speed Reserve, stratified for ASCVD.

| **Moderator** | **Estimated Coefficient** | **95% CI** | **P-value** | **M-ATE_0_** | **M-ATE_1_** |
| --- | --- | --- | --- | --- | --- |
| **No history of ASCVD (n=4118)** |  |  |  |  |  |
| ACEi | 5.14 | (0.10, 10.2) | 0.05 | -3.58 | 1.55 |
| Aspirin | 5.98 | (1.55, 10.4) | 0.008 | -4.00 | 1.98 |
| **History of ASCVD (n=1401)** |  |  |  |  |  |
| ACEi | 1.08 | (-4.27, 6.43) | 0.69 | 3.23 | 4.31 |
| Aspirin | 0.73 | (-4.42, 5.88) | 0.78 | 2.00 | 2.73 |

Supplementary Table 6. Linear regression results for the PS-weighted linear model for outcome Gait Speed Reserve, stratified for cognition.

| **Moderator** | **Estimated Coefficient** | **95% CI** | **P-value** | **M-ATE_0_** | **M-ATE_1_** |
| --- | --- | --- | --- | --- | --- |
| **Normal cognition (n=2362)** |  |  |  |  |  |
| ACEi | 6.48 | (0.70, 12.3) | 0.03 | -4.70 | 1.79 |
| Aspirin | 6.92 | (1.93, 11.9) | 0.006 | -4.71 | 2.21 |
| **Cognitive impairment (n=2808)** |  |  |  |  |  |
| ACEi | 1.48 | (-3.15, 6.11) | 0.53 | -0.13 | 1.35 |
| Aspirin | 3.09 | (-1.12, 7.31) | 0.15 | -0.77 | 2.32 |
